# Patterns of Firearm Ownership and Opinions on Firearm Policies Among Adults in California

**DOI:** 10.1001/jamanetworkopen.2020.12096

**Published:** 2020-07-31

**Authors:** Julia P. Schleimer, Rocco Pallin, Garen J. Wintemute, Amanda Charbonneau, Nicole Kravitz-Wirtz

**Affiliations:** 1 Violence Prevention Research Program, Department of Emergency Medicine, University of California, Davis, School of Medicine, Sacramento; 2 University of California Firearm Violence Research Center, Sacramento

## Abstract

This survey study assess whether patterns in firearm ownership are associated with opinions on firearm safety among adults in California.

## Introduction

Few studies^1^ have examined variation in firearm policy opinions among firearm owners, even though these policies, which may target specific firearms or behaviors, may not affect all owners equally. In addition, the confluence of individuals’ firearm-related practices and motivations may reflect differences among owners that are unmeasured (eg, concern for safety) or unobservable (eg, culture) and that are associated with policy opinions.

With use of a state-representative survey of California adults, a previous study^2^ identified 5 latent classes of firearm ownership based on numbers and types of firearms owned, the primary reason for ownership, firearm storage, loaded handgun carrying behavior and motivations, and high-capacity ammunition magazine ownership. In this study, we assessed whether those patterns were associated with variation in opinions on 3 selected firearm policies.

## Methods

Data for this survey study were from the 2018 California Safety and Well-being Survey administered from September 14, 2018, to October 12, 2018.^3^ The California Safety and Well-being Survey was approved by the University of California, Davis Institutional Review Board. Respondents received a standard informed consent page online. This study followed the American Association for Public Opinion Research (AAPOR) reporting guideline.

We examined support for 3 firearm policy proposals according to previously identified^2^ latent classes of ownership: owners of ≥5 firearms, including handguns and long guns (class 1); owners of 1 long gun for a reason other than protection (class 2); owners of 1 handgun for protection (class 3); owners of 2 to 4 firearms, including at least 1 for protection (class 4); and owners of ≥5 firearms, including assault weapons, and high-capacity magazines (class 5) (Table 1). Classes represent otherwise unobservable subgroups. Two of the firearm proposals concerned a ban on possession of high-capacity (>10 rounds) ammunition magazines, which was approved by voters in California in 2016 but since has been challenged in federal court.^4^ One proposal was for an amnesty to relinquish magazines, and the other was for a buyback. The third proposal, under consideration in the 2019 to 2020 California legislative session,^5^ would impose a time-limited firearm prohibition on individuals with multiple recent convictions for driving under the influence (DUI). We used χ^2^ tests (2-sided *P* < .05) to examine differences in policy opinions by class. All percentages are weighted.

**Table 1.** Defining Characteristics of 5 Latent Classes of Firearm Owners, 2018 California Safety and Well-being Survey^a^

Characteristic	Class 1	Class 2	Class 3	Class 4	Class 5
Firearms owned. No.	≥5	1	1	2-4	≥5
Types of firearms owned	HG and LG	LG only	HG only	HG and LG	AW and other firearms
Any firearm owned primarily for protection against people	No	No	Yes	Yes	Yes
Firearm storage^b^	Most secure	Storage practices varied	Moderately secure	Least secure	Least secure
Loaded handgun carrying	No	No	No	No	Yes, primarily for protection against people
High-capacity ammunition magazine ownership	No	No	No	No	Yes

Abbreviations: AW, assault weapon; HG, handgun; LG, non–assault-type long gun.

^a^ Adapted from Schleimer et al (2019)^2^ with permission from BMJ Publishing Group Limited.

^b^ Least secure indicates that the firearm was unlocked and loaded; moderately secure, locked and loaded or unlocked and unloaded; and most secure, locked and unloaded.

## Results

Of 5232 individuals invited to participate, 2558 (49%) completed the survey. Most owners (136 [31.0%; 95% CI, 24.9%-37.9%]) belonged to class 1, and the fewest (28 [8.2%; 95% CI, 4.8%-13.6%]) belonged to class 5. A total of 234 firearm owners (51.0%; 95% CI, 43.9-58.2) supported the amnesty proposal; 249 (55.1%; 95% CI, 47.8-62.2), the buyback proposal; and 248 (49.9%; 95% CI, 42.7-57.0), the DUI proposal. Support for the amnesty proposal varied significantly across classes (Table 2); it was highest among the single long gun owner (class 2; 74 [67.1%; 95% CI, 52.0%-79.3%]) and single handgun owner (class 3; 64 [62.3%; 95% CI, 48.8%-74.2%]) classes and lower among the multiple firearm owner classes. Support was lowest (8 adults; 34.3% [95% CI, 14.4%-61.9%]) and opposition highest (14 adults; 53.0% [95% CI, 27.5%-76.9%]) among individuals in class 5, the multiple firearm owner class uniquely likely to own high-capacity magazines and assault-type weapons. Support for the buyback proposal was similar to that for amnesty. Support for a DUI-based prohibition ranged from 41.2% (95% CI, 19.1%-67.5%) in class 5 to 62.4% (95% CI, 48.4%-74.6%) in class 3.

**Table 2.** Support for Firearm Violence Prevention Proposals Among Firearm Owners by Patterns of Ownership, 2018 California Safety and Well-being Survey^a^

Characteristic	Unweighted No. (weighted %) [95% CI]					
	Class 1	Class 2	Class 3	Class 4	Class 5	Total
Total	136 (31.0) [24.9-37.9]	107 (25.6) [19.6-32.6]	95 (20.8) [16.1-26.6]	63 (14.4) [9.7-20.8]	28 (8.2) [4.8-13.6]	429 (100)
Amnesty for high-capacity magazines^b^^,^^c^						
Support	60 (39.2) [28.4-51.2]	74 (67.1) [52.0-79.3]	64 (62.3) [48.8-74.2]	28 (41.0) [22.4-62.6]	8 (34.3) [14.4-61.9]	234 (51.0) [43.9-58.2]
Oppose	41 (32.0) [21.2-45.2]	20 (18.2) [10.2-30.2]	18 (22.9) [13.8-35.6]	19 (36.8) [19.4-58.5]	14 (53.0) [27.5-76.9]	112 (29.0) [22.8-36.1]
Do not know	33 (28.0) [17.8-41.2]	12 (14.3) [5.8-31.0]	13 (14.7) [7.5-26.9]	16 (22.2) [11.0-39.8]	6 (12.7) [4.6-30.8]	80 (19.7) [14.5-26.1]
Buyback for high-capacity magazines^d^						
Support	70 (45.8) [34.1-57.9]	72 (68.5) [53.7-80.3]	67 (69.0) [55.8-76.7]	32 (44.0) [25.0-65.0]	8 (32.8) [13.4-60.6]	249 (55.1) [47.8-62.2]
Oppose	34 (22.4) [14.9-32.3]	15 (10.5) [5.4-19.7]	16 (15.7) [8.9-26.2]	17 (35.7) [18.5-57.6]	16 (56.5) [30.5-79.3]	98 (22.7) [17.4-29.0]
Do not know	29 (30.7) [18.9-45.9]	19 (20.5) [10.6-35.9]	12 (15.3) [7.7-28.2]	14 (20.3) [9.6-37.9]	4 (10.8) [3.3-29.6]	78 (21.8) [16.0-28.9]
DUI-based prohibition^e^						
Support	69 (42.8) [31.5-54.9]	65 (47.9) [33.7-62.4]	65 (62.4) [48.4-74.6]	38 (55.5) [35.0-74.4]	11 (41.2) [19.1-67.5]	248 (49.9) [42.7-57.0]
Oppose	41 (34.4) [23.4-47.3]	25 (34.3) [21.1-50.5]	16 (19.6) [11.3-31.9]	16 (36.1) [18.6-58.1]	13 (32.1) [13.8-56.5]	111 (31.3) [24.9-38.6]
Do not know	25 (22.3) [12.6-36.4]	17 (17.8) [8.5-33.5]	14 (18.0) [9.1-32.4]	9 (8.4) [3.8-17.7]	4 (26.7) [7.2-63.0]	69 (18.6) [13.2-25.6]
All^c^						
Support	38 (26.7) [17.7-38.2]	43 (34.2) [22.2-48.6]	50 (48.2) [35.1-61.6]	22 (34.8) [17.1-58.0]	5 (26.3) [9.0-56.2]	158 (34.2) [27.8-41.3]
Oppose	13 (8.7) [4.6-15.9]	2 (3.7) [0.9-14.1]	6 (8.1) [3.4-18.2]	10 (27.0) [11.6-51.2]	9 (23.5) [9.5-47.4]	40 (11.2) [7.4-16.5]
Do not know	7 (5.4) [2.2-12.6]	5 (10.8) [3.3-29.8]	5 (7.1) [2.3-19.5]	1 (0.9) [0.1-6.4]	2 (6.3) [1.2-26.3]	20 (6.6) [3.5-11.8]
Mixed	74 (57.8) [45.8-69.0]	56 (50.8) [36.3-65.3]	34 (36.6) [24.6-50.4]	30 (37.3) [21.6-56.2]	12 (44.0) [20.2-71.0]	206 (47.5) [40.4-54.7]

Abbreviation: DUI, driving under the influence.

^a^ Data from the survey are from Kravitz-Wirtz et al.^3^ Columns may not sum to total because of missing values. Firearm owners were assigned to the class in which they had the highest probability of belonging according to posterior probabilities of class membership estimated by the latent class analysis model. Unweighted numbers reflect counts of respondents; survey-weighted percentages and 95% CIs incorporate California Safety and Well-being Survey weights.

^b^ Respondents were asked: “Would you support or would you oppose an amnesty program that allows people to turn in ammunition magazines that hold more than 10 bullets, no questions asked?”

^c^ *P* < .05.

^d^ *P* = .002. Respondents were asked: “Would you support or would you oppose a buyback program that allows people to turn in ammunition magazines that hold more than 10 bullets, no questions asked, in exchange for a small payment or reward?”

^e^ Respondents were asked: “Would you support or would you oppose a law that prevents someone from buying a gun for 5 years if they have had 2 or more DUI convictions in 5 years?”

## Discussion

This was the first study, to our knowledge, to assess differences in firearm policy support according to patterns of firearm ownership. We observed between-class differences in support for high-capacity ammunition magazine proposals but not for a DUI-based prohibition.

Support for high-capacity magazine amnesty and buyback was lower in the multiple firearm owner classes. Opposition was highest among the class likely to own high-capacity magazines and assault-type weapons. Because opposition may influence compliance, particularly for policies in which the impetus for action resides with firearm owners, our findings provide additional context for the mixed results of past research on high-capacity magazine buyback programs.^6^ The small size of this class (n = 28) necessitates further research.

A previous study found^1^ that 41.4% of high-capacity magazine owners supported the amnesty proposal, which was greater support than observed in this study for class 5 (34.3%). This finding suggests heterogeneity among high-capacity magazine owners, not all of whom were assigned to class 5, captured in part by these latent patterns.

Limitations include potential nonresponse, recall, selection, and social desirability biases. Results from this study may be specific to California. Because of the small sample, these findings are suggestive rather than conclusive. Nevertheless, the diversity of firearm owners’ opinions in this survey study challenges the notion that firearm owners are a monolithic group. Continued research on variation among owners may advance firearm policy development and evaluation.
